# Supplementary material for: Energy landscape underlying spontaneous insertion and folding of an alpha-helical transmembrane protein into a bilayer
Source: Nat Commun. 2018 Nov 23;9:4949. doi: 10.1038/s41467-018-07320-9 (PMC6251876; doi:10.1038/s41467-018-07320-9)
Supplement: Supplementary file 1 — Supplementary Information [file 41467_2018_7320_MOESM1_ESM.pdf]

Supplementary Information for "Energy landscape underlying spontaneous insertion and folding of an alpha-helical transmembrane protein into a bilayer"

Wei Lu[1,3,*], Nicholas P. Schafer[1,2,*], and Peter G. Wolynes[1,2,3,4,†]

[1]Center for Theoretical Biological Physics, Rice University, Houston, TX, USA
[2]Department of Chemistry, Rice University, Houston, TX, USA
[3]Department of Physics, Rice University, Houston, TX, USA
[4]Department of Biosciences, Rice University, Houston, TX, USA
[*]These authors contributed equally to this work.
[†]Please address correspondence to pwolynes@rice.edu.

October 16, 2018

Supplementary Figures

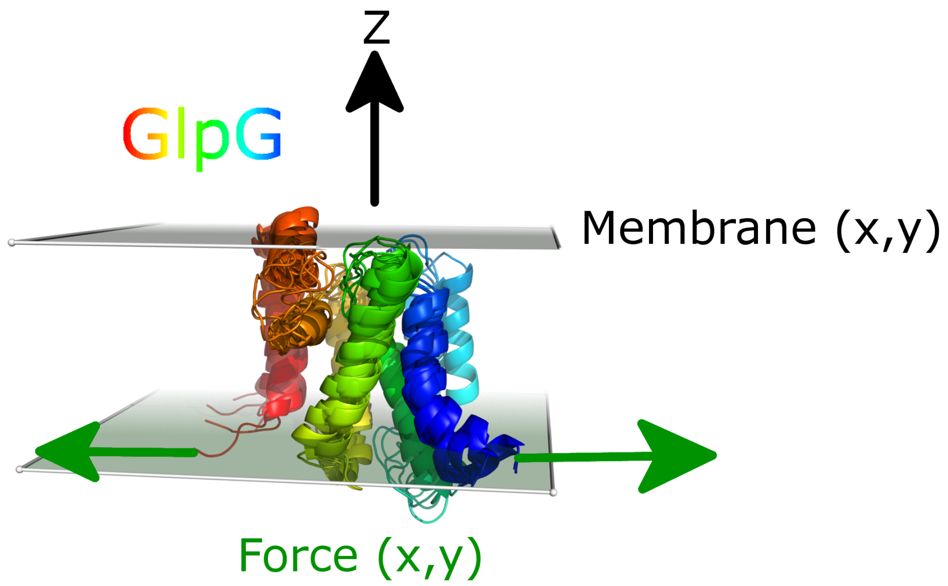

Supplementary Figure 1: Schematic diagram of the simulation setup. GlpG is placed in an implicit membrane that is flat, stretches infinitely in the (x, y) plane, and has a finite thickness in the z direction. The z direction is parallel to the membrane normal. Umbrella sampling combined with temperature replica exchange is used to sample conformations that have a wide range of end-to-end distances.

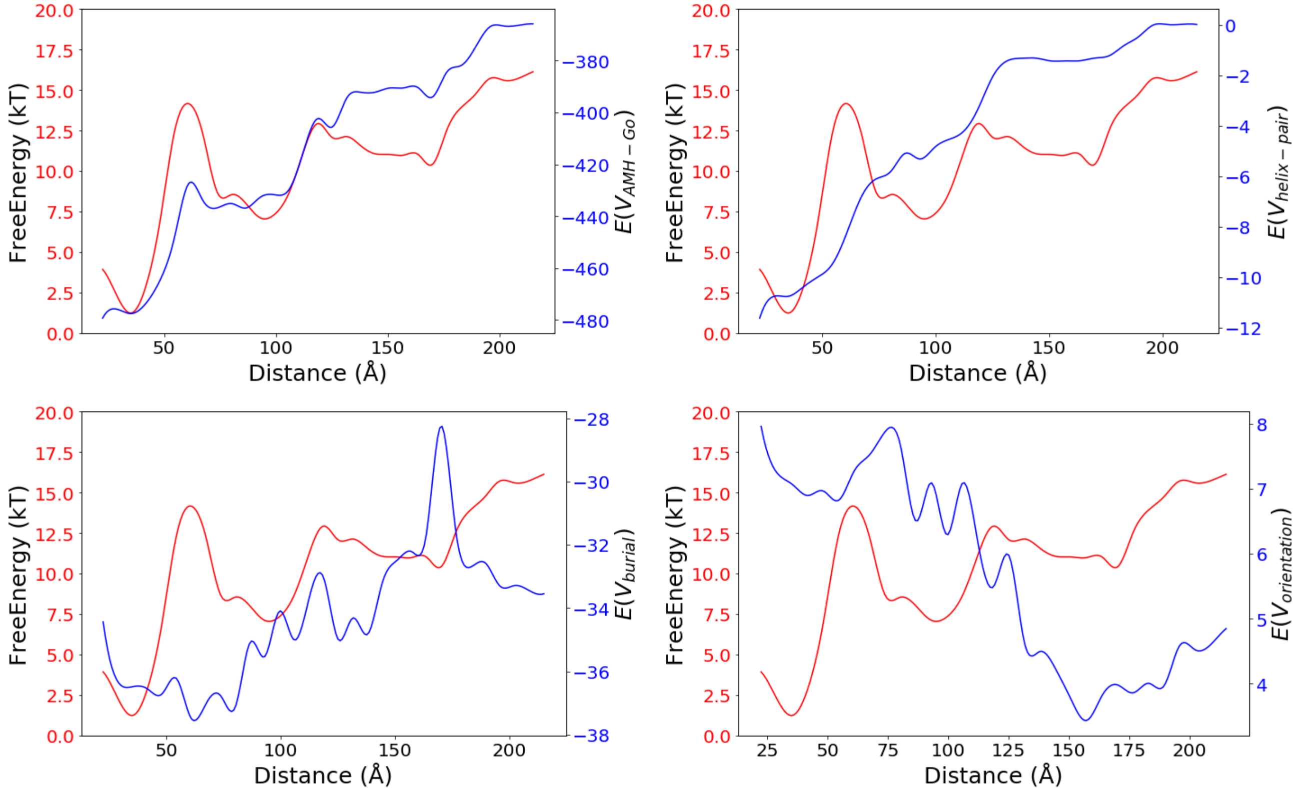

Supplementary Figure 2: Expectation values of the structure-based and implicit membrane energies along the inferred folding pathway. The expectation values of four energy terms (V_{AMH-Go}, $V_{helix-pair}$, V_{burial}, and $V_{orientation}$) are shown as a function of the end-to-end distance, D, along the inferred folding pathway at low applied force. The expectation values are plotted in blue, and the free energy along the folding pathway is shown in red.

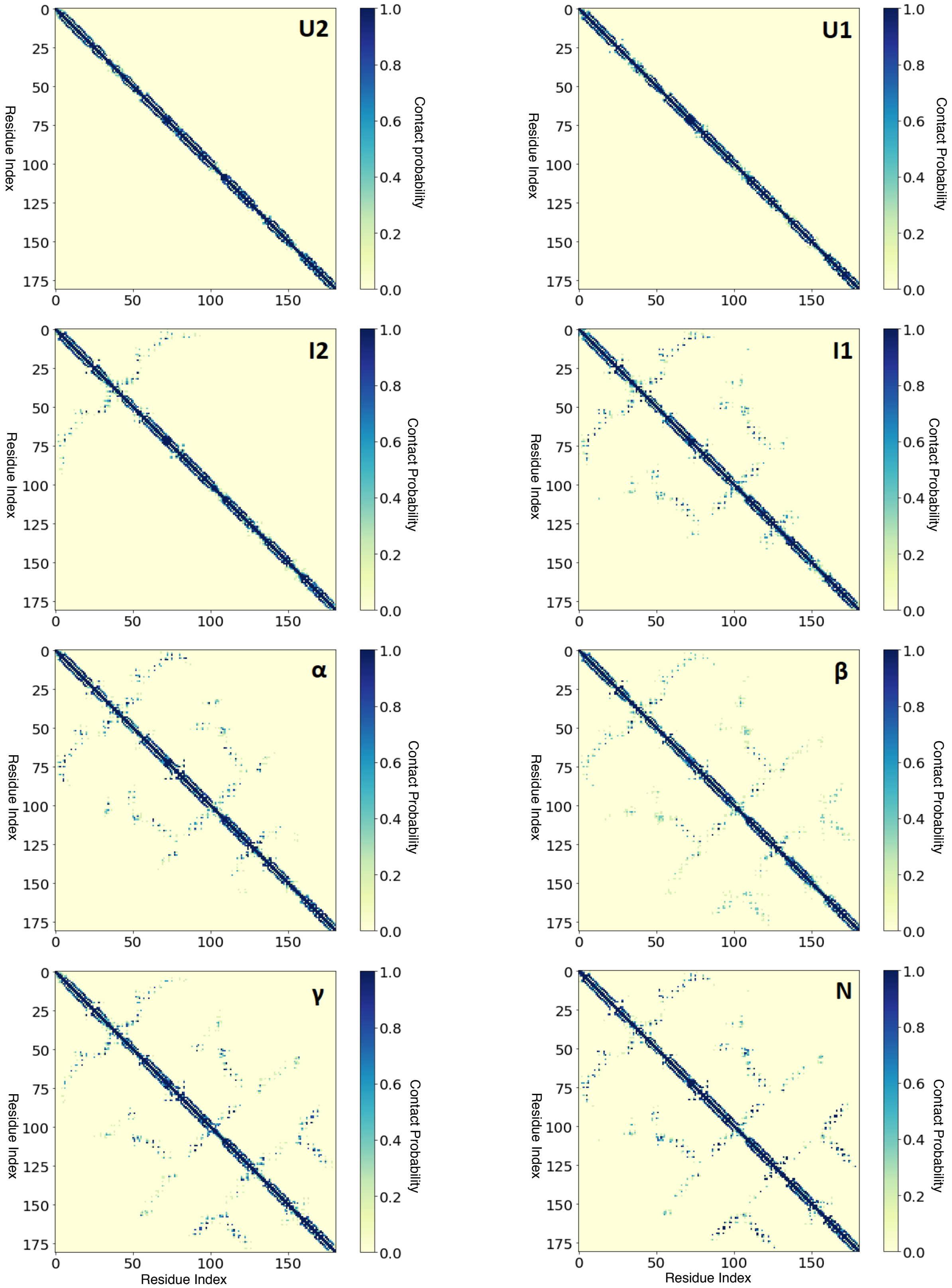

Supplementary Figure 3: Average contact maps for the structural ensembles listed in Supplementary Table 1. Contacts are defined based on a 7$\AA$ $C_\beta - C_\beta$ cutoff (C_α for glycine). The color indicates the frequency at which a particular contact is formed in the indicated ensemble.

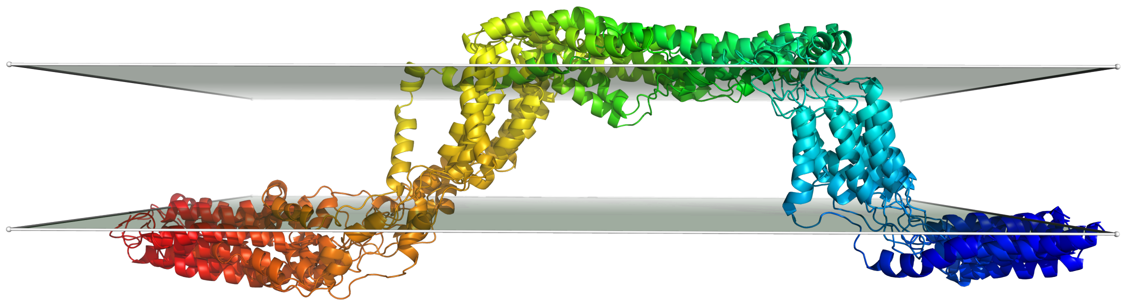

Supplementary Figure 4: Some highly extended structures with a non-native orientation of transmembrane helices with respect to the membrane. An ensemble of GlpG structures with TM2 (shown in yellow) and TM5 (shown in light blue) embedded in the membrane. This ensemble has an average D value of $220\mathring{A}$ and an average Z value of $-3\mathring{A}$ (see Fig. 3 in the main text). The structure of GlpG is colored according to sequence index from red (N-terminal, TM1) to blue (C-terminal, TM6). Several representative structures are aligned and overlayed. Translucent panels are shown that indicate the locations of the upper and lower bilayer interfaces. For clarity, all of the structures have been aligned, but only a single location of the upper and lower bilayer interfaces are shown.

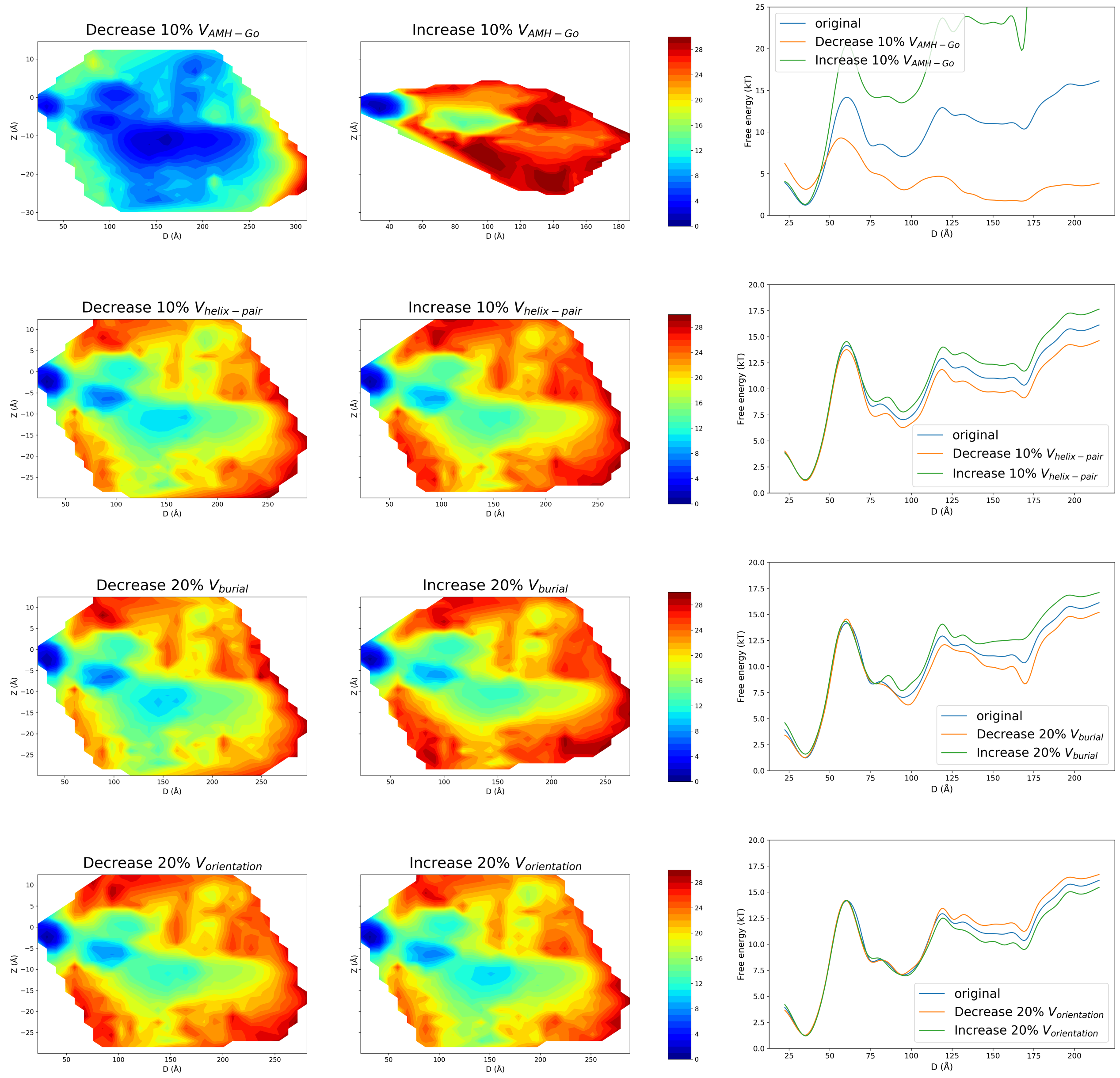

Supplementary Figure 5: Free energy landscapes and profiles obtained while perturbing the structure-based protein and implicit membrane energy terms. For each energy term (V_{AMH-Go}, $V_{helix-pair}$, V_{burial}, and $V_{orientation}$), the two-dimensional free energy landscapes as a function of D and Z are shown while decreasing (left) or increasing (middle) the strength of the term. The one-dimensional free energy profiles for the perturbed and unperturbed models are compared on the right.

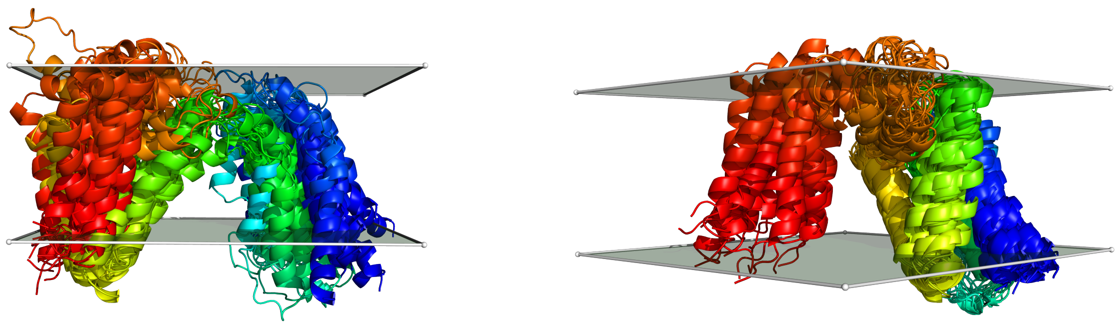

Supplementary Figure 6: Native-basin structures of GlpG that overlap the $I1 \rightarrow N$ transition state in (D, Z) coordinate space. (Left) An ensemble of GlpG structures with TM1-3 and TM4-6 separately inserted and folded and the interface between these two subdomains broken. This ensemble has an average D value of $64\mathring{A}$ and an average Z value of $-2.5\mathring{A}$. (Right) An ensemble of GlpG structures with TM1 and TM2-6 separately inserted and folded and the interface between these two subdomains broken. This ensemble has an average D value of $57\mathring{A}$ and an average Z value of $-2.5\mathring{A}$. For reference, the $I1 \rightarrow N$ transition state shown in Fig. 2 in the main text has an average D of $61\mathring{A}$ and an average Z of $-3.5\mathring{A}$. The structure of GlpG is colored according to sequence index from red (N-terminal, TM1) to blue (C-terminal, TM6). For each state, several representative structures are aligned and overlayed. Translucent panels are shown that indicate the locations of the upper and lower bilayer interfaces. For clarity, all of the structures have been aligned, but only a single location of the upper and lower bilayer interfaces are shown.

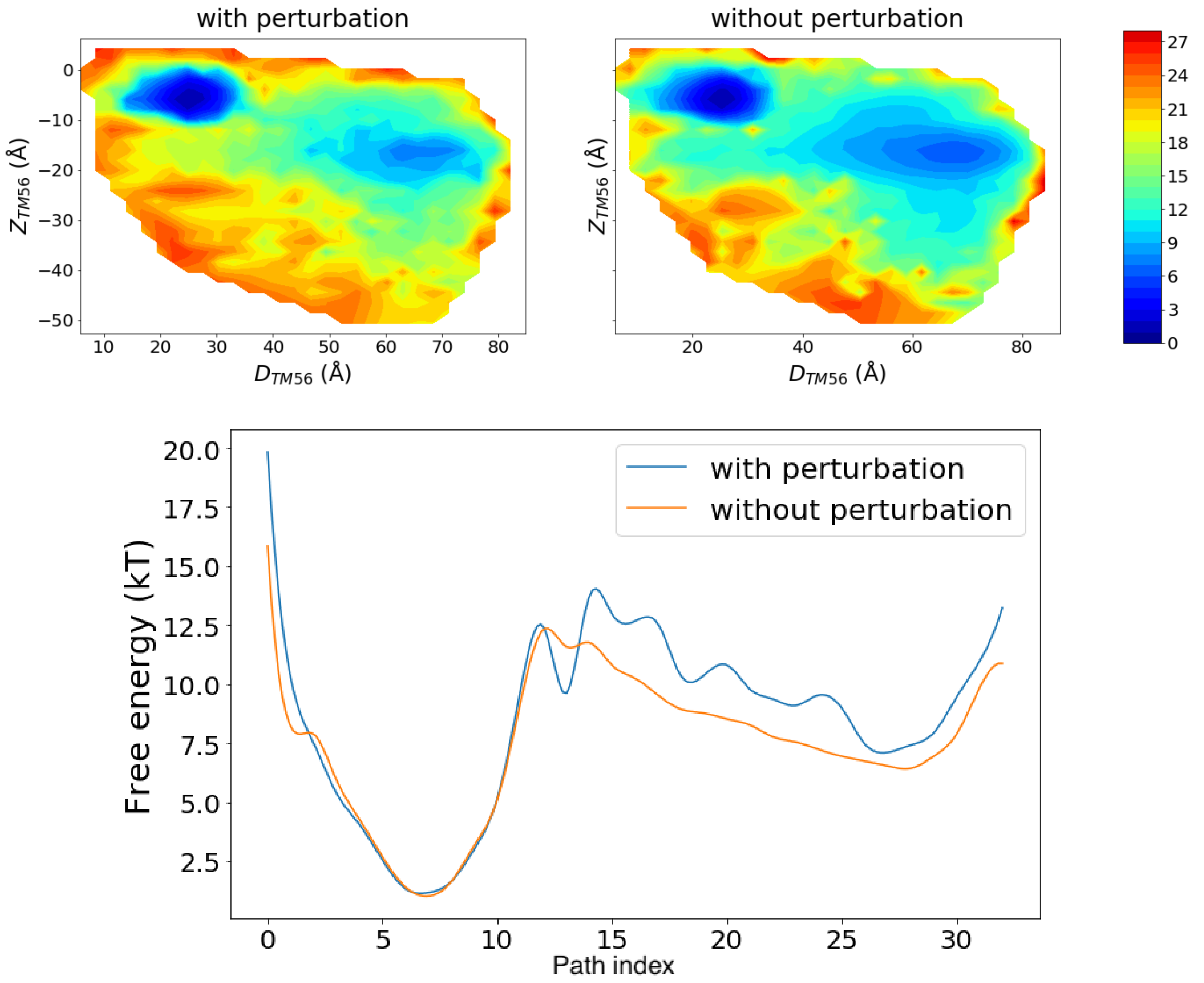

Supplementary Figure 7: Free energy landscapes and profiles with and without the perturbation to the strength of contacts within TM1-4 plotted as a function of D_{TM5-6} and Z_{TM5-6}. (Top) Two-dimensional free energy landscapes as a function of D_{TM5-6} and Z_{TM5-6} with and without the application of the 20% perturbation to the strength of the contacts within helices TM1-4. (Bottom) The free energy profiles along the low free energy pathways across the landscapes in the Top panel. In the bottom panel, the free energy profiles are shown as a function of path index, where low path indices correspond to low D (folded and inserted) states and high path indices correspond to relatively high D (partially folded) states.

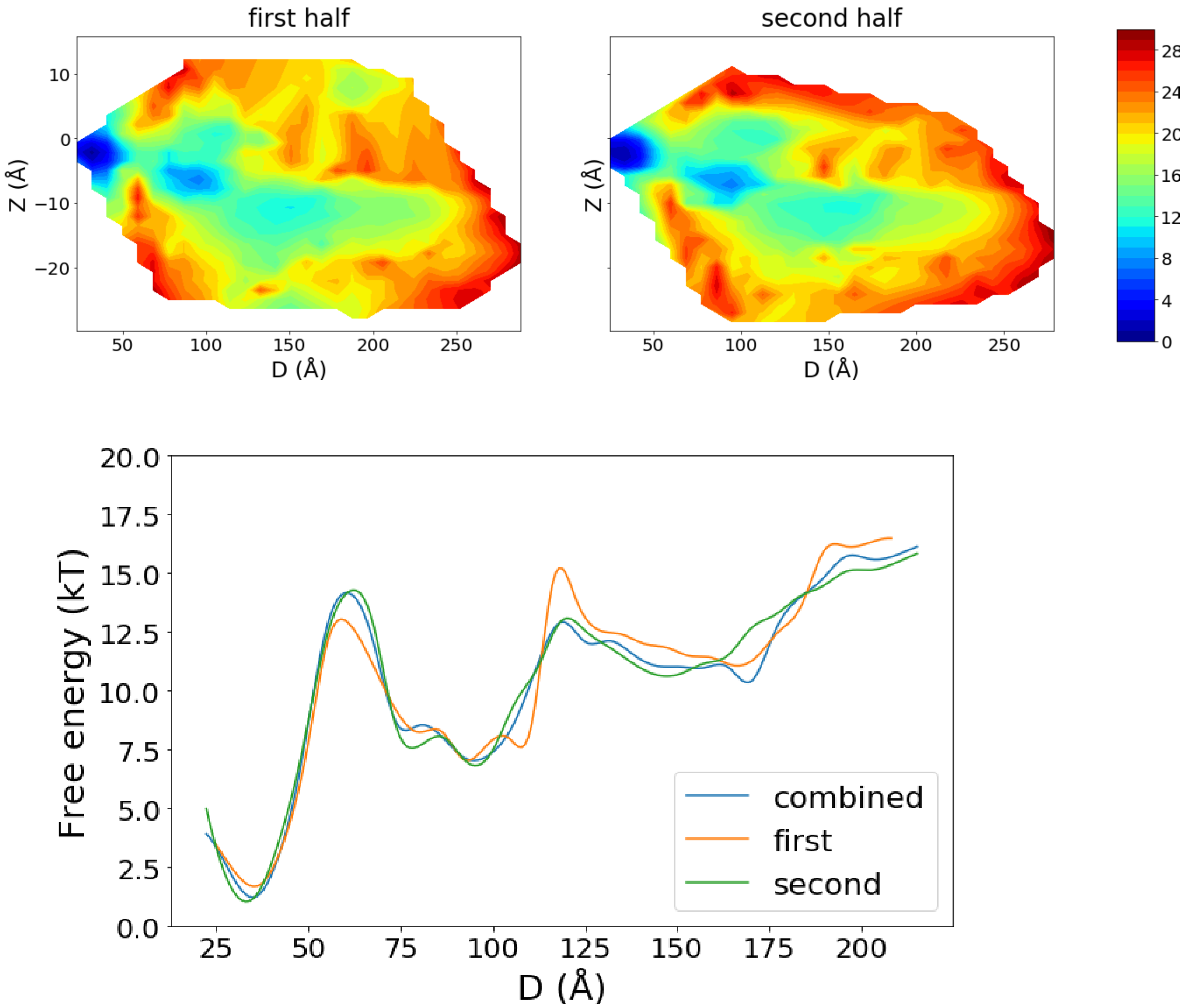

Supplementary Figure 8: A comparison of free energy landscapes and profiles obtained by using either the first half or the second half of the data as input to the pyMBAR algorithm. (Top) Two-dimensional free energy landscapes as a function of D and Z. (Bottom) Free energy profiles along the inferred folding pathways using either the first half (orange), the second half (green), or the complete data set (blue).

Supplementary Tables

Ensemble	D ($\AA$)	Z ($\AA$)	Q	D_{TM5-6} ($\AA$)	Z_{TM5-6} ($\AA$)
U2	271.92±7.33	-18.89±1.80	0.09±0.00	73.53±3.32	-17.13±1.18
U1	226.45±2.65	-12.60±1.45	0.09±0.01	73.45±2.34	-17.01±0.66
I2	145.80±6.41	-9.66±0.82	0.16±0.01	67.45±4.91	-16.10±1.33
I1	83.98±7.36	-6.58±0.85	0.42±0.02	61.94±6.30	-17.44±2.68
γ	67.08±1.55	-5.51±0.37	0.46±0.03	53.40±5.05	-13.78±1.07
β	60.19±1.87	-3.91±0.59	0.41±0.06	40.16±17.73	-10.14±3.53
α	53.38±1.06	-3.14±0.58	0.59±0.03	27.51±3.53	-5.60±1.34
N	34.94±2.84	-2.55±0.35	0.75±0.03	26.52±2.10	-5.45±0.24

Supplementary Table 1: A summary of structural characteristics of ensembles of structures along the folding and unfolding pathway of GlpG in the presence of the membrane. The ensemble labels are the same as those used in the main text. D and Z are the end-to-end distance and the average z-value of the C_α atoms in GlpG, respectively. Q is the fraction of pairwise distances between the C_α atoms in GlpG that are within $1\AA$ of their corresponding value in the crystal structure of GlpG (PDB ID: 2XOV). D_{TM5-6} is the end-to-end distance of TM5-6. Z_{TM5-6} is the average z-value of the C_α atoms in TM5-6. For each collective variable and each ensemble, the average and standard deviation of the collective variable computed over randomly selected structures from within the ensemble is given.

Supplementary Notes

1 Simulation and analysis overview

A schematic diagram showing the simulation setup is shown in Supplementary Figure 1. In the experiments of Min et al., bicelles are used as a bilayer-mimicking environment. Bicelles are self-assembling aggregates of lipid and detergent molecules that consist of a patch of lipid bilayer, which is similar to the in vivo environment of transmembrane proteins, that is then encircled by a belt of detergent molecules. In single molecule force spectroscopy experiments, the problem of membrane protein aggregation is solved by performing measurements on single molecules effectively at extremely low concentrations. The application of force is used to shift the equilibrium between the folded and unfolded states without the need for adding chemical denaturants that can perturb membranes. By measuring the end-to-end distance changes during unfolding events along with measuring the sensitivity of rates to the magnitude of the applied force, some structural aspects of the folding mechanism can also be inferred. In the simulations, the folding of GlpG is studied in the presence of an implicit membrane. A sampling scheme employing umbrella sampling along the end-to-end distance combined with temperature replica exchange simulations was used to obtain an equilibrated set of conformations with end-to-end distances ranging from distances compatible with the folded state to distances consistent with a fully extended state. Free energy landscapes as a function of the end-to-end distance and the overall degree of insertion into the membrane are then calculated at various values of the applied force. The folding and unfolding pathways are inferred by finding low free energy paths to and from the folded state. Finally, the structural mechanism of folding and unfolding is inferred by examining the structural ensembles present in the free energy basins through which the low free energy path proceeds. All of the above steps are described in detail in the Methods section.

2 Energy term expectation values and free energy profiles obtained while perturbing individual energy terms

To help us understand the influence of the various energy terms in the structure-based protein model and implicit membrane model on the folding mechanism, we have plotted the expectation values of the energy terms along the inferred folding pathway at low applied force. These plots are shown in Supplementary Figure 2. V_{AMH-Go}, the structure-based term that stabilizes native contacts, decreases monotonically throughout most of the folding process but increases at the location of the free energy barrier between $I1$ and N. This increase in energy is consistent with the partial unfolding noted in the main text on the basis of examining the structural ensembles along the $I1 \rightarrow N$ folding transition. V_{AMH-Go} is the only energy term that exhibits an obvious local maximum at the free energy barrier between $I1$ and N. $V_{helix-pair}$, the generic lipid-mediated pairwise interaction between transmembrane helices, decreases monotonically throughout folding as helices become buried in the membrane. V_{burial}, the amino acid type-dependent term governing the favorability of burial in the membrane, exhibits a peak at the location of a local free energy minimum in the $I2$ basin. At this same local minimum in the free energy, V_{AMH-Go} is at a local minimum, highlighting the possibility of frustration between V_{AMH-Go} and the implicit membrane energy function. There is not an obvious peak in V_{burial} during the $I1 \rightarrow N$ transition, consistent with the relatively short and hydrophobic nature of L5 between TM5 and TM6. $V_{orientation}$ is relatively low in the $I2$ basin and rises during the $I2 \rightarrow I1$ transition due to the fact that GlpG's transmembrane helices, when folded into the native conformation, are not perfectly parallel to the membrane normal.

As another means of investigating the influence of the individual energy terms in the combined protein-implicit membrane forcefield on the folding mechanism of GlpG, we have systematically perturbed the strength of each energy term and plotted the resulting free energy landscapes and free energy profiles along the folding pathway. The resulting landscapes and profiles are shown in Supplementary Figure 5. Increasing the strength of V_{AMH-Go} increases the height of the barrier between N and $I1$ in the unfolding direction. Increasing the strength of $V_{helix-pair}$ decreases the height of the barrier between $I1$ and N in the folding direction. Increasing the strength of V_{burial} decreases the height of the already small barrier between $I2$ and $I1$ in the folding direction, whereas increasing the strength of $V_{orientation}$ has the opposite effect.

3 Tables and contact maps summarizing structural characteristics of the ensembles along the folding and unfolding pathway of GlpG in the presence of a membrane

To more fully characterize the ensembles of structures that were identified along the folding and unfolding pathways of GlpG, we have computed the averages and standard deviations of several structural order parameters for these ensembles. The results of these calculations are given in Supplementary Table 1. We have also computed the average contact maps for these structural ensembles, and the results of this analysis are shown in Supplementary Figure 3.

In Supplementary Table 1, the ensembles along the folding pathway of GlpG are listed from top (unfolded states) to bottom (native state). D, the end-to-end distance, is seen to decrease monotonically throughout the folding process as Z increases, indicating that folding and insertion into the membrane are coupled. Q, a measure of similarity to the crystal structure, increases at every step during the process except when going from γ to β. This decrease in similarity to the native structure when going from γ to β is consistent with the unfolding at the transition state, β, noted in the main text and

with the increase in the V_{AMH-Go} energy shown in Supplementary Figure 2. D_{TM5-6}, the end-to-end distance of TM5-6, decreases slowly on average throughout the first few steps of folding and then drops dramatically during the final refolding transition. The standard deviation of D_{TM5-6} is very large at the transition state, β, consistent with the idea that folding and insertion of TM5-6 is the rate-limiting step of refolding at low applied force. Similarly, Z_{TM5-6}, the average z-value of C_α atoms in TM5-6, indicates that the helices remain near the bilayer interface ($\approx -17\mathring{A}$) during the first few folding steps and are then inserted during the final folding transition.

The average contact maps for the ensembles listed in Supplementary Table 1 are given in Supplementary Figure 3. In $U1$ and $U2$, only local helical contacts are formed. In $I2$, contacts between TM1 and TM2 have formed, as well as some contacts within L1, the large loop between TM1 and TM2. In $I1$, contacts between the TM1-4 have formed, but contacts between TM5-6 and the rest of the protein are absent. There are very few contacts gained during the first part of the final refolding transition ($I1 \to \gamma$). At the transition state, β, contacts between TM5-6 and the rest of the protein have begun to form and contacts within TM1-4 are found at a lower frequency than they were in γ, indicating that the N-terminal part of GlpG undergoes a "loosening". This unfolding is consistent with the decrease in Q value seen in Supplementary Table 1, the increase in V_{AMH-Go} seen in Supplementary Figure 2, and the variations in the structures in the β ensemble discussed in the main text. Completion of folding ($\beta \to \gamma \to N$) corresponds to consolidation of the contact pattern seen in β.

4 Some highly extended structures with non-native orientations of transmembrane helices with respect to the membrane

At high values of the applied force, there are some highly extended conformations of GlpG that are relatively low in free energy and have native-like Z values. On the basis of their D and Z values alone, these structures might appear to be candidates for the unfolded state in the two-stage picture of membrane protein folding. However, as can be seen in Supplementary Figure 4, these structures, in fact, have non-native orientations of the transmembrane helices with respect to the membrane. The high degree of insertion into the membrane (as indicated by the native-like Z values) comes from having several entire transmembrane helices on the opposite bilayer interface from the termini.

5 Native-basin structures that overlap the $I_1 \to N$ refolding transition state in (D, Z) coordinate space

Several ensembles of structures were found to overlap the $I1 \to N$ transition state in (D, Z) coordinate space (Supplementary Figure 6). When the free energy landscape of GlpG's insertion and folding into a membrane is plotted as a function of D and Z and the folding pathway is inferred by looking for a low free energy path in (D, Z) space, the presence of these ensembles leads to apparent downhill folding and insertion at low values of the applied force, which would contradict the experimental observations in [6]. Preferential stabilization of TM1-4 over TM5-6 to a degree that is quantitatively consistent with the steric trapping measurements made in [5] raises the free energy of the ensembles in Supplementary Figure 6 such that the folding pathway along D and Z is clarified and a significant barrier between the native state and a partially folded intermediate state ($I1$) is apparent in (D, Z) space. The intermediate state $I1$ has an average end-to-end distance that is quantitatively consistent with the sum of the distances to the transition state inferred by force spectroscopy [6] and is therefore a good candidate for the starting point for refolding at low force. In contrast, the ensembles shown in Supplementary Figure 6 have a much shorter end-to-end distance and are therefore not good candidates for the starting point for refolding at low force.

6 Free energy profile plotted using reaction coordinates that focus on the $I1 \to N$ folding transition

In the main text, D and Z are used as reaction coordinates for plotting the free energy landscapes. D is a natural reaction coordinate to use because it corresponds to the experimental observable in the force spectroscopy experiments of Min et al. [6]. Z is another natural reaction coordinate to use because the extent of burial of the protein into the membrane is arguably the single most important thermodynamic order parameter describing folding and insertion that is not directly an experimental observable in the force spectroscopy experiments. In most cases, D and Z together separate well the low free energy basins ranging from the highly extended states to the folded state. For the near-native states, however, several ensembles in the native basin overlap the transition state for the $I1 \to N$ transition, leading to apparent downhill folding at low force that would contradict one of the main experimental observations from force spectroscopy. To clarify the folding pathway in (D, Z) space, the interactions within TM1-4 of GlpG were preferentially stabilized by 20% using perturbation theory. To help us understand the influence of this perturbation on the $I1 \to N$ transition, we have plotted the free energy landscapes both with and without applying the perturbation using order parameters that allow us to focus on the $I1 \to N$ transition. D_{TM5-6} is the end-to-end distance of TM5-6 and Z_{TM5-6} is the average z-value of the C_α atoms within TM5-6.

The structures shown in Supplementary Figure 6 overlap the $I1 \to N$ transition state in (D, Z) space. However, because these structures differ from the fully native state only by partial unfolding of TM1-4 and therefore have native-like values of D_{TM5-6} and Z_{TM5-6}, these ensembles do not overlap the $I1 \to N$ transition state in (D_{TM5-6}, Z_{TM5-6}) coordinate space. The free energy profiles shown in Supplementary Figure 7 indicate that, both with and without the application of

the perturbation, there are two dominant free energy basins corresponding to $I1$ and N. Furthermore, application of the perturbation does not significantly change the relative free energies of $I1$, N, and the transition state between $I1$ and N.

7 Test of the convergence of sampling used to compute free energy profiles

As is described in the Methods section, 20 million steps worth of data were used to compute the free energy profiles that are shown in the main text. To test the convergence of sampling used to compute the free energy profiles, we recomputed the free energy landscapes as a function of D and Z using either only the first 10 million steps worth of data or only the last 10 million steps worth of data and compared the predicted free energy profiles along the inferred folding pathways to those obtained by combining both halves of the data, which is the same set of data that was used to compute the profiles presented in the main text. The result of this analysis is shown in Supplementary Figure 8. Both the first half and the second half of the data yield free energy profiles that are consistent with the discussion of the results given in the main text, and the profile obtained using the second half of the data set is highly similar to that for the combined data set.

Supplementary References

[1] Bobby L Kim, Nicholas P Schafer, and Peter G Wolynes. Predictive energy landscapes for folding α-helical transmembrane proteins. *Proceedings of the National Academy of Sciences*, 111(30):11031–11036, 2014.

[2] Ha H Truong, Bobby L Kim, Nicholas P Schafer, and Peter G Wolynes. Predictive energy landscapes for folding membrane protein assemblies. *The Journal of chemical physics*, 143(24):243101, 2015.

[3] Nicholas P Schafer, Ha H Truong, Daniel E Otzen, Kresten Lindorff-Larsen, and Peter G Wolynes. Topological constraints and modular structure in the folding and functional motions of glpg, an intramembrane protease. *Proceedings of the National Academy of Sciences*, 113(8):2098–2103, 2016.

[4] Patrick Lagüe, Martin J Zuckermann, and Benoit Roux. Lipid-mediated interactions between intrinsic membrane proteins: dependence on protein size and lipid composition. *Biophysical Journal*, 81(1):276–284, 2001.

[5] Ruiqiong Guo, Kristen Gaffney, Zhongyu Yang, Miyeon Kim, Suttipun Sungsuwan, Xuefei Huang, Wayne L Hubbell, and Heedeok Hong. Steric trapping reveals a cooperativity network in the intramembrane protease glpg. *Nature chemical biology*, 12(5):353, 2016.

[6] Duyoung Min, Robert E Jefferson, James U Bowie, and Tae-Young Yoon. Mapping the energy landscape for second-stage folding of a single membrane protein. *Nature chemical biology*, 11(12):981, 2015.
